# The combined impact of AI and VR on interdisciplinary learning and patient safety in healthcare education: a narrative review

**DOI:** 10.1186/s12909-025-07589-7

**Published:** 2025-07-11

**Authors:** Emmanuel Aoudi Chance

**Affiliations:** VID Specialized University, Ulriksdal 10, Bergen, Norway

**Keywords:** Artificial intelligence (AI), Virtual reality (VR), Healthcare education, Interdisciplinary learning, Patient safety, Adaptive learning pathways, Team-based simulations

## Abstract

**Introduction:**

The increasing integration of Artificial Intelligence (AI) and Virtual Reality (VR) in healthcare education offers innovative ways to enhance collaborative learning and improve patient safety. This narrative review examines the synergistic impact of AI-powered virtual reality (VR) simulations, such as those used in surgical training and patient communication, on knowledge acquisition, clinical skill development, and collaborative competencies among healthcare students. It also explores long-term knowledge retention, ethical considerations within virtual scenarios, and the psychological impact of high-stakes simulations on learner resilience.

**Methods:**

This review is distinguished by its exhaustive literature search, which spanned PubMed, CINAHL, Scopus, Google Scholar, and other pertinent databases, to identify studies published between 2005 and 2024. The distinct focus on AI and VR interventions in healthcare education, particularly those with outcomes related to interdisciplinary learning or patient safety, distinguishes this review. Data were thematically analyzed across domains, including adaptive learning, technical skill development, teamwork, patient safety, and ethics.

**Results:**

The findings of this review carry significant practical implications. Five key themes emerged: adaptive learning (*n* = 17), immersive skill development (*n* = 10), teamwork enhancement (*n* = 10), patient safety (*n* = 18), and ethical considerations (*n* = 21). These themes underscore the potential of AI and VR in healthcare education. AI-driven adaptive systems enabled personalized VR training, enhancing engagement and knowledge retention. Real-time AI feedback during simulations improved decision-making in safe, controlled environments. Interdisciplinary team simulations enhanced communication and collaboration, which are crucial for effective clinical care. Ethical modules embedded in VR scenarios promoted moral reasoning. Several studies also reported increased learner confidence in performing clinical procedures following VR training, suggesting enhanced preparedness for practice.

**Conclusion:**

The integration of AI and VR holds the potential to revolutionize healthcare education, fostering personalized, immersive, and ethically informed learning. These technologies enhance technical proficiency and equip students with the complex demands of modern clinical practice. Strategic implementation can contribute to error reduction, improved patient outcomes, and a culture of safety. However, the journey is not over. Continued research is crucial for assessing the long-term outcomes and cost-effectiveness of these educational innovations, as well as for keeping pace with the rapidly evolving field of AI and VR in healthcare education.

**Supplementary Information:**

The online version contains supplementary material available at 10.1186/s12909-025-07589-7.

## Introduction

The rapid advancements in Artificial Intelligence (AI) and Virtual Reality (VR) are transforming healthcare education, providing dynamic, interactive, and immersive learning experiences that surpass traditional training methods. A range of AI models are commonly applied in healthcare and education contexts [1, 2] (Table 7). Historically, medical education relied on didactic instruction and static simulations, which, while valuable, often lacked the adaptability and realism necessary for comprehensive training in clinical decision-making and interdisciplinary collaboration. Increasingly, these traditional approaches are being supplemented—or even replaced—by AI- and VR-powered simulations that better align with real-world demands and enhance essential aspects of patient safety [3]. Given the complexity of healthcare delivery and the critical role of interdisciplinary teamwork, there is an urgent need for training tools that simulate realistic, team-based scenarios fostering collaboration, communication, and patient-centered care [4].

A significant advantage of AI in healthcare education is its ability to create adaptive learning pathways tailored to each student’s progress, offering immediate and specific feedback that helps students address weaknesses and build on strengths, accelerating skill acquisition [5]. VR, by contrast, enables hands-on, high-fidelity simulations that replicate a broad spectrum of clinical scenarios, from complex surgical procedures to patient-centered care in high-stakes emergency settings [6]. By combining AI’s adaptability with VR’s immersive qualities, students gain vital experience through repeated practice, real-time decision-making, and collaborative scenarios—all within a safe, controlled environment that poses no risk to actual patients [6, 7].

### The established impact: what we already know

Existing research provides key insights into the combined impact of AI and VR on interdisciplinary learning outcomes and patient safety in healthcare education:

#### Enhanced skill development through immersive simulation

The integration of AI and VR in healthcare education creates immersive simulations that enable students to practice complex clinical procedures in a controlled environment. This combination fosters hands-on learning by simulating real-life scenarios without the associated risks, allowing students to make decisions, learn from mistakes, and receive instant AI-driven feedback. Research by Pottle (2019) has shown that students who engage with AI-augmented VR simulations demonstrate improved procedural skills and critical thinking, both of which are essential for maintaining patient safety. These simulations not only provide interdisciplinary exposure but also promote teamwork and communication skills, which are critical in real-world settings and in ensuring patient safety [8].

#### Improved ethical and clinical Decision-Making

AI-enhanced VR simulations enable students to navigate complex ethical and clinical decision-making scenarios by presenting realistic situations that require prioritization, ethical consideration, and prompt responses. Studies such as those by Liu et al. (2023) and Skulmowski (2023) suggest that exposing students to ethical dilemmas within virtual reality (VR) simulations strengthens their moral reasoning and ability to apply ethical principles in patient care [9, 10]. This capability is vital for patient safety, as ethical dilemmas often arise in critical care, where decisions must be patient-centered and balanced against clinical urgency. The role of AI and VR in fostering ethical decision-making underscores the moral responsibility in patient care, enhancing the audience’s understanding of the importance of these technologies.

#### Increased confidence and reduced error rates in practice

VR, coupled with AI-driven feedback, has significantly reduced learners’ error rates and increased their confidence and competence, particularly in high-stakes, error-prone procedures. According to a study by Anthamatten (2024), students trained with AI-VR simulations experience fewer errors when transitioning to real clinical practice compared to those trained through traditional methods [11]. This reduction in errors not only boosts confidence but also enhances patient outcomes, as healthcare providers who feel competent are less likely to make mistakes under pressure. The role of AI in reducing error rates provides reassurance of patient safety, making the audience feel more confident in the potential of these technologies.

### Recent innovations and emerging developments

Recent advancements further highlight the transformative potential of AI and VR in healthcare education, introducing innovative approaches to interdisciplinary learning and patient safety:

#### Adaptive learning algorithms in VR simulations

AI-driven adaptive learning algorithms are now integrated into VR simulations to provide a more personalized and responsive educational experience. These algorithms, powered by AI, assess each student’s progress in real time, identifying strengths and areas for improvement and then adjusting the complexity of scenarios based on their current competence. For instance, students performing well in routine simulations may encounter more complex, high-stress scenarios, challenging them to expand their skills. This innovation promotes efficient learning, ensuring that students acquire fundamental and advanced clinical skills at a pace tailored to their learning curves [12–14].

#### Cross-Disciplinary, Real-Time collaborative simulations

A new advancement in interdisciplinary training utilizes VR for real-time collaborative simulations that connect students from different healthcare disciplines within a single virtual environment. This approach allows nursing, medical, and allied health students to practice teamwork and communication skills as if they were physically working together on patient cases. Using AI, these VR platforms facilitate realistic interactions, enabling students to work through tasks simultaneously and learn from one another’s contributions. Research by Dhar et al. (2023) highlights that this form of interdisciplinary, real-time VR collaboration enhances communication skills and situational awareness, both of which are essential for effective patient care [15].

#### Integration of AI for predictive error analysis and risk assessment

Cutting-edge VR platforms now utilize AI to analyze predictive errors and assess potential risks to student performance. By analyzing data from repeated simulation runs, AI can identify patterns in student behavior that may indicate a predisposition to specific errors or unsafe practices, such as hesitating during critical decision-making moments or misinterpreting patient data. This information is used to tailor feedback, advise students on areas for improvement, and highlight how these habits may impact patient outcomes. According to recent studies by Alowais et al. (2023) and Rony et al. (2024), this predictive capability enhances patient safety preparedness. It helps students develop a more proactive approach to minimizing errors and optimizing care [16, 17].

Given the complexity of healthcare delivery and the critical role of interdisciplinary teamwork, there is an urgent need for training tools that simulate realistic, team-based scenarios fostering collaboration, communication, and patient-centered care [4]. Despite the potential of AI and VR, research on their combined impact on interdisciplinary learning outcomes and patient safety remains limited. Most studies have explored the effects of either AI or VR on specific objectives, such as procedural accuracy or knowledge retention [18]. However, understanding how these technologies work together could reveal synergies that enhance skill transfer, team-based collaboration, and clinical decision-making—critical elements for patient safety in complex, interdisciplinary care settings [19]. Addressing this gap, the present narrative review explores the combined impact of AI and VR in healthcare education, synthesizing current literature to assess their potential to foster effective interdisciplinary learning and prioritize patient-centered, safety-oriented care.

Despite the growing interest and the clear benefits of individual AI or VR applications, a comprehensive narrative review specifically examining their combined impact on *interdisciplinary learning outcomes* and *patient safety* in healthcare education remains a significant gap in the current literature. Addressing this crucial gap, particularly amidst rapidly evolving healthcare technologies and the urgent need for interdisciplinary practice readiness, this review systematically synthesizes existing evidence to illuminate how AI-enhanced VR simulations can foster effective team-based learning, enhance clinical decision-making, and directly contribute to a more safety-oriented healthcare workforce, thereby guiding educators in leveraging these powerful tools for future training.

### Aims

This narrative review aims to bridge the existing research gap by examining the dual application of AI and VR in healthcare education, with a focus on their combined impact on interdisciplinary learning outcomes and patient safety. Specifically, it evaluates how these technologies support adaptive, high-stakes simulation training and explores implications for long-term knowledge retention and the ethical dimensions of simulated learning experiences. Through this analysis, the review seeks to provide a comprehensive understanding of AI and VR as an integrated approach to preparing healthcare professionals for the complexities of real-world patient care.

### Significance of this study

This study addresses an urgent need in healthcare education for innovative training approaches that enhance learning and prioritize patient safety. As healthcare becomes increasingly interdisciplinary, with professionals from various fields collaborating in high-stakes environments, training students in collaborative decision-making and patient-centered care is paramount [20, 21]. The combined application of AI and VR represents a transformative step toward meeting this need, offering adaptive and realistic simulations that prepare students for complex clinical scenarios without compromising patient safety.

By examining the effects of AI and VR on interdisciplinary learning and patient safety, this study contributes to the expanding body of research on educational technology in healthcare. It highlights how AI’s adaptability and VR’s immersive qualities can support individualized skill development and real-time feedback, fostering a safer and more effective learning environment. The findings offer valuable insights for educators, policymakers, and institutions aiming to implement or optimize technology-driven training methods, ultimately benefiting future healthcare professionals and their patients. This study highlights the broader importance of integrating advanced technologies into healthcare education, equipping students with the skills and confidence necessary to enhance patient outcomes and foster a culture of safety in clinical practice.

### Novelty and contribution

This study represents a transformative initiative in the field of healthcare education, as it leverages the potential of Artificial Intelligence (AI) and Virtual Reality (VR) in a groundbreaking manner. By utilizing AI to construct dynamic, responsive scenarios and VR to provide an immersive, lifelike environment, we are set to revolutionize clinical skills, collaborative practices, and safety awareness in healthcare settings. This innovative approach establishes a more immersive, realistic, and responsive educational environment, enabling healthcare professionals to engage with complex, simulated scenarios that closely resemble real-world situations.

The study introduces a pioneering framework that integrates AI and VR, systematically assessing their influence on interdisciplinary teamwork—a critical component in patient care that has not been sufficiently examined in technology-enhanced education. Unlike traditional simulation training, the AI-VR model is uniquely designed to adapt to real-time participant decisions and interactions, providing dynamic feedback that challenges and hones decision-making and teamwork skills. This adaptive feedback loop instills confidence in the AI-VR model, enabling quick and informed responses to patient safety risks and more effectively preparing healthcare teams for high-stakes clinical environments.

Moreover, this research makes a significant contribution to the evolving field of patient safety in healthcare education by quantitatively and qualitatively evaluating the effects of AI-VR integration on critical safety competencies. These include situational awareness, error prevention, and communication across professional boundaries. The potential impact of our findings is substantial, as they provide compelling evidence of the potential for AI and VR to reinforce safety-oriented learning. This study lays the groundwork for future research into scalable, interdisciplinary, technology-driven educational models, instilling hope for the future of patient safety in healthcare education.

### Main research question

How does combining Artificial Intelligence (AI) and Virtual Reality (VR) impact interdisciplinary learning outcomes and patient safety in healthcare education?

#### Additional questions

How do AI-driven adaptive learning pathways within VR simulations influence healthcare students’ skill acquisition, long-term knowledge retention, and interdisciplinary collaboration?

What ethical and psychological considerations, including student resilience and emotional well-being, must be addressed when implementing high-stakes, AI-enhanced VR simulations in healthcare training to support patient safety?

What technical and institutional challenges exist in developing, standardizing, and sustaining AI-VR applications in healthcare education, and how can cross-institutional efforts contribute to best practices and consistent educational outcomes?

## Methods

This study follows a structured review approach adapted from Ferrari. (2015), covering the period from 2005 to 2024. Narrative reviews are valuable for identifying patterns, addressing specific questions, and organizing complex topics in fields of growing research interest. According to Jackson et al., an effective review requires a well-organized synthesis of previous research to provide a coherent analysis [22]. This narrative review aims to deepen understanding of the combined impacts of AI and VR on interdisciplinary learning and patient safety in healthcare, to bridge gaps in the existing literature, and to highlight areas for further exploration.

### Search strategy

A multi-step search strategy was designed to capture relevant literature on AI and VR in healthcare education, structured as follows:

Straightforward research questions and objectives were established to focus the search and ensure alignment with the study’s aims. Critical academic databases relevant to the research topic were selected, including PubMed, CINAHL, Scopus, Google Scholar, Medline (Ovid), EBSCOhost, Education Resources Information Center (ERIC), Education Source, and Web of Science, to ensure comprehensive coverage of sources. Keywords and related synonyms were identified to target specific topics, such as “AI in healthcare education,” “VR simulations,” “interdisciplinary learning,” “patient safety,” and “clinical decision-making.” Boolean operators (AND, OR, NOT) were applied to combine terms, creating search strings such as (“Virtual Reality” OR “AI technology”) AND (“Patient safety” OR “Clinical decision-making”) AND (“Healthcare education” OR “Interdisciplinary learning”). Filters limited results to articles from the last 10 years, peer-reviewed studies, and specific types such as systematic reviews and experimental studies. Grey literature, including conference proceedings and reports from healthcare organizations, was consulted for additional insights.

Articles were organized using Mendeley, allowing for efficient reference tracking and management. Each step of the search process, including databases, keywords, and applied filters, was recorded for transparency and replicability. Relevant data were extracted and synthesized by the study’s research questions and aims.

### Inclusion and exclusion criteria

Inclusion and exclusion criteria were applied to select articles most relevant to the study’s questions and objectives, with a strong emphasis on practical application. The criteria included relevance to healthcare education, the use of AI and VR in interdisciplinary learning or patient safety, and studies published within the past decade. Peer-reviewed journal articles were included, excluding non-peer-reviewed content, and studies that focused on theoretical frameworks without practical application were also excluded. After an initial search, *(N = 5939)* articles were retrieved. Duplicates were removed, and some conference papers were excluded, resulting in *(N = 118)* articles. Further screening based on title, abstract, and relevance, along with evaluating methodological rigor, led to a final selection of *(N = 76)* articles reviewed in depth (Appendix A).

### Data analysis

Data analysis was conducted on a final set of *82* articles, selected based on the inclusion criteria. The process followed three main stages:

The quality of the studies was evaluated using PRISMA (Preferred Reporting Items for Systematic Reviews and Meta-Analyses) guidelines to ensure relevance to AI and VR in healthcare education and adherence to methodological standards [22]. Critical information was extracted, encompassing aspects such as study design, impacts on patient safety, learning outcomes, and the roles of AI and VR in interdisciplinary training. Data was organized into themes, including “clinical decision-making improvements,” “development of interdisciplinary skills,” and “patient safety enhancements.” These themes were analyzed to identify patterns and gaps, with synthesized findings reflecting these insights.

The data were further organized into categories and subcategories to provide a clear overview of the thematic findings (Table 1). Key themes included the influence of VR on clinical simulations, the role of AI in providing personalized feedback, and the combined effects on patient safety.

This analysis provided a comprehensive understanding of the influence of AI and VR on interdisciplinary learning and patient safety in healthcare, highlighting both their strengths and areas for future research.

The layout (Table 1) provides a clear overview of the analysis of the qualified article, highlighting the main themes and findings in each row, along with relevant information.

Table 2 provides a structured approach to examining findings across the main themes of adaptive learning, immersive skill development, teamwork, patient safety, and ethical considerations in healthcare training that utilizes AI and VR.

Table 2 includes additional authors for each subcategory, presenting a comprehensive view of adaptive learning through AI-driven VR in healthcare education.

Table 3 highlights immersive skill development through virtual reality (VR) in healthcare education, with additional authors listed for each subcategory.

Table 4 highlights interdisciplinary teamwork in healthcare education through the use of VR and AI, with additional authors listed for each subcategory.

Table 5 presents key findings on Patient Safety Preparedness in healthcare education using VR and AI, with additional authors listed for each subcategory.

Table 6 highlights the key findings on the ethical and psychological impact of VR and AI applications in healthcare education, with additional authors listed for each subcategory.

## Trustworthiness of this narrative study

The trustworthiness of this narrative study, *Exploring the Combined Impact of Artificial Intelligence and Virtual Reality on Interdisciplinary Learning Outcomes and Patient Safety in Healthcare Education*, is reinforced by its rigorous methodological approach and transparent reporting. The study follows a systematic search strategy guided by clearly defined inclusion and exclusion criteria to ensure comprehensive coverage of the relevant literature. Each phase of the review process, from literature selection to data extraction, is documented in line with the Preferred Reporting Items for Systematic Reviews and Meta-Analyses (PRISMA) guidelines, ensuring transparency and replicability.

Further enhancing credibility, this review integrates data from peer-reviewed journals and studies selected based on relevance to the research questions. The study presents a coherent and balanced perspective on the impact of AI and VR on interdisciplinary learning outcomes and patient safety, employing thematic analysis to synthesize the findings. The methodological rigor and transparent synthesis contribute to a reliable and valuable addition to the field, offering insights that can inform educational practices and patient safety initiatives in healthcare.

## Findings

The analysis presents findings (*N* = 76) across the main themes of adaptive learning *(n1 = 17)*, immersive skill development *(n2 = 10)*, teamwork *(n3 = 10)*, patient safety *(n4 = 18)*, and ethical considerations in healthcare training using AI and VR (*n5 = 21)*.

### Adaptive learning

The use of Artificial Intelligence (AI) to create adaptive learning pathways within Virtual Reality (VR) simulations holds significant promise for enhancing healthcare education, as it enables a personalized and responsive approach to training (Table 2). In adaptive learning, AI technology continuously assesses learners’ strengths and weaknesses, tailoring educational content accordingly. In VR, each simulation can be customized to focus on areas where students may need more practice while progressively increasing the complexity as they demonstrate competency [9, 23, 24].

AI-driven personalizations within VR simulations are particularly relevant in healthcare training, where practical skills, accuracy, and the ability to respond under pressure are essential. Students who engaged in AI-adapted VR simulations showed improved procedural accuracy and confidence compared to traditional simulation methods [25–27]. This approach breaks away from the ‘one-size-fits-all’ model in education, enabling students to develop their competencies individually and ultimately leading to improved knowledge retention and skill acquisition [28]. Moreover, real-time feedback from AI systems in VR not only corrects mistakes as they occur but also helps students understand the rationale behind best practices, fostering a deeper understanding of patient safety protocols and emphasizing the importance of continuous learning and improvement in healthcare [29, 30].

An additional benefit of AI-driven adaptive learning is its potential to enhance long-term retention of skills. According to Elendu et al. (2024), Halkiopoulos and Gkintoni (2024), and Zhai et al. (2021), personalized feedback and adaptive learning are associated with higher levels of memory retention in complex clinical scenarios. The studies suggest that when students receive instant, tailored feedback on specific actions during VR-based simulations, they are more likely to retain and apply that information in actual clinical settings. These findings are particularly significant for high-stakes healthcare fields, where the ability to recall and execute procedures correctly can directly impact patient outcomes, underscoring the crucial role of healthcare professionals in improving patient care [6, 31, 32].

Integrating artificial intelligence (AI) and virtual reality (VR) into healthcare education can revolutionize training and learning experiences. AI-enhanced VR applications can create immersive, realistic simulations for medical students, clinicians, and healthcare professionals to hone their skills. However, the development, maintenance, and long-term support of these systems present several significant technical challenges that institutions must address to ensure their continued efficacy, reliability, and scalability. These challenges include resource demands, system integration, data security, and ongoing software updates [5, 33, 34].

Adaptive learning in AI-powered VR simulations represents a shift toward a more learner-centered approach in healthcare education. By aligning simulation training with individual learning needs, adaptive AI not only enhances students’ procedural skills but also empowers them to handle complex patient care scenarios with greater confidence and precision, inspiring a new generation of healthcare professionals [6, 35–39].

### Immersive skill development

The immersive qualities of Virtual Reality (VR) create a unique environment for healthcare students to develop critical clinical skills in a risk-free yet realistic setting (Table 3). VR’s ability to replicate high-fidelity clinical scenarios enables students to practice procedures and patient interactions with a degree of detail and immersion that traditional methods often cannot match [40]. VR simulations facilitate experiential learning by engaging students deeply with complex clinical tasks, ranging from surgical techniques to emergency response, thus promoting hands-on skill development crucial in high-stakes medical environments [8].

One of VR’s primary strengths lies in its ability to simulate high-stakes scenarios that healthcare students may rarely encounter during their training, yet are essential for ensuring patient safety in real-world settings. Jacobs & Maidwell-Smith, 2022; Rushton et al., 2020; Trevi et al., 2024 explored the use of VR for simulating cardiac arrest situations. They found that students engaged in these immersive simulations demonstrated increased confidence and accuracy in performing cardiopulmonary resuscitation (CPR). Their study highlights how VR’s realistic, 360-degree environments enable repeated exposure to critical procedures, allowing students to build competency through practice and correction without endangering patient lives. This form of repetitive, high-fidelity practice has been linked to higher levels of clinical skills or performance, as students who regularly participate in VR simulations tend to show significant improvements in skill retention and application [41–43].

Additionally, VR’s immersive nature not only aids in the acquisition of technical skills but also enhances students’ capacity for decision-making under pressure. In scenarios that replicate real-life patient interactions, students make quick, high-impact decisions, such as diagnosing conditions or selecting treatment paths, thereby fostering their clinical judgment and situational awareness. VR simulations improved decision-making skills and procedural confidence among nursing students as they encountered and managed realistic challenges within the simulated environment [9, 44].

VR’s immersive properties contribute to a training environment where healthcare students can repeatedly engage in complex clinical situations, develop a deeper understanding of patient care practices, and gain practical experience without the risks associated with real-life training. By building these competencies in a safe, controlled environment, VR offers a valuable avenue for developing critical skills transferable to clinical settings, helping ensure that healthcare professionals are well-prepared for the demands of patient-centered care.

### Interdisciplinary teamwork

Integrating Virtual Reality (VR) and Artificial Intelligence (AI) in healthcare education offers promising opportunities to enhance interdisciplinary teamwork—an essential skill for ensuring effective patient care (Table 4). Collaboration across disciplines, including nursing, medicine, and allied health professions, is critical for holistically managing patient cases. VR simulations offer an immersive platform for students from diverse fields to participate in team-based scenarios, where they must communicate effectively and make joint decisions under realistic conditions. AI further strengthens this environment by dynamically adjusting simulations based on team interactions, prompting students to address real-time challenges collaboratively [45–47].

One of the notable benefits of team-based VR simulations is their ability to improve communication and decision-making among healthcare students. VR-based team simulations markedly improved students’ ability to communicate effectively, even in high-pressure situations, such as acute trauma cases [48]. Students trained in VR simulations gained a better understanding of each team member’s role, ultimately leading to more coordinated and faster decision-making. VR’s immersive nature can emulate the stress and urgency of real-world clinical settings, providing a practice ground for the skills required to operate in interdisciplinary teams under pressure [49].

Moreover, interdisciplinary VR simulations have been shown to cultivate role specialization awareness, a critical aspect of effective teamwork in healthcare. Understanding and respecting each discipline’s contributions enables healthcare professionals to work effectively and efficiently, making informed patient-care decisions. Kim et al. (2024), S. Liaw et al. (2020), and Qiao et al. (2021) found that students who participated in VR simulations involving diverse team roles reported a deeper appreciation of interprofessional collaboration. Students honed their skills and gained insight into how other professionals contribute to patient outcomes, which is vital for fostering mutual respect and reliance in clinical settings [4, 50, 51].

Furthermore, AI-enhanced VR environments enable simulations to present unique, unplanned challenges—such as sudden patient deterioration or unexpected complications—that require team members to quickly reassess and adapt their strategy. This adaptability mirrors real-life healthcare scenarios, where flexibility and clear communication are essential for positive outcomes. VR simulations designed for interdisciplinary teams promote adaptive expertise, enabling students to learn how to adjust their communication and strategies in response to evolving clinical situations [51–53]. This adaptability is essential for patient safety and security, as it encourages future healthcare professionals to remain coordinated and responsive to changes, thereby preventing adverse events.

It is important to recognize the pivotal role of healthcare professionals, educators, and policymakers in shaping the future of healthcare and public health education through the integration of immersive technologies (VR and AR) and AI. These technologies can enhance learning, improve outcomes, and promote standardization across institutions. The need for establishing clear guidelines and fostering cross-institutional collaboration is a recurring theme in these works. Standardized guidelines and cross-institutional studies are essential for establishing the best practices in the use of AI and VR technologies in healthcare education. These initiatives help ensure safety, consistency, and high-quality learning outcomes across institutions. As AI and VR continue to reshape healthcare training, these frameworks will remain crucial in promoting the widespread adoption of practical, evidence-based approaches that can ultimately improve healthcare delivery and patient outcomes [6, 8, 54].

Utilizing AI and VR for interdisciplinary teamwork training in healthcare education offers a realistic and engaging approach for students to develop and refine the collaborative skills essential for patient-centered care. By simulating team-based clinical experiences, VR and AI prepare students to navigate the complexities of modern healthcare environments, where effective teamwork is critical to ensuring patient safety and quality outcomes.

### Patient safety preparedness

Virtual Reality (VR) combined with Artificial Intelligence (AI) is invaluable in enhancing patient safety preparedness within healthcare education (Table 5). In VR-based simulations, students can practice complex and high-stakes procedures in a controlled environment, where mistakes or errors can be corrected without compromising patient safety. AI-driven real-time feedback in these VR settings enables learners to identify errors and modify their actions on the spot, fostering safer practices and improving clinical competence over time. Students using AI-enhanced VR simulations significantly reduced error rates when they later performed procedures in natural clinical settings. The ability to practice and refine skills repeatedly in VR simulations without risk to patients has contributed substantially to the safe transition from learning to practice [55–58].

VR’s capacity to simulate ethical scenarios and decision-making also prepares students for the moral challenges they may encounter in patient care. Healthcare providers often face ethical dilemmas that require quick decisions, and VR simulations provide a safe space for students to explore these situations. VR simulations incorporating AI-driven feedback on ethical choices helped students recognize and assess risks more effectively, leading to better-informed decisions. This exposure to ethical complexities allows students to practice making balanced, patient-centered decisions in high-stakes scenarios, enhancing their readiness for real-life practice [59–61].

Furthermore, VR-based training has been linked to greater confidence among students when approaching clinical procedures and patient interactions, which can contribute positively to patient safety. Nursing students who underwent VR simulation training reported higher confidence levels in performing procedures and managing patient crises than those trained through traditional methods [62–64]. This increased confidence is crucial, as studies have shown that healthcare professionals who feel confident in their skills are less likely to make errors under pressure. In this way, VR fosters a readiness for practice that directly translates into safer, more effective patient care.

In addition, real-time error recognition and correction in AI-augmented VR simulations allow students to build a reflexive approach to patient care. Simulations can replicate various “what-if” scenarios, giving students insight into the potential consequences of their actions. Students trained with VR experienced reduced critical safety incidents when entering clinical practice, as they had developed a heightened awareness of potential risks [65–68]. This proactive approach to patient safety—cultivated through repeated exposure to error recognition and response training in VR—equips students with the mental frameworks necessary to anticipate, identify, and manage safety risks in real-world settings.

By providing students with realistic, practice-intensive experiences, VR and AI significantly improve patient safety preparedness. These tools support the development of error-averse, patient-centered practices that enhance student confidence and prioritize the well-being of future patients. In this way, VR and AI are shaping a new generation of healthcare providers better equipped to deliver safe, high-quality care from the start of their careers.

### Enhanced clinical decision-making skills

Artificial Intelligence (AI) and Virtual Reality (VR) in healthcare education enhance students’ clinical decision-making skills, enabling them to make informed choices in complex scenarios with confidence and accuracy (Table 6). By simulating realistic patient interactions and medical scenarios, VR provides a hands-on learning environment where students can apply diagnostic reasoning and treatment planning without facing real-life consequences. AI-driven feedback within these simulations provides tailored guidance that helps learners understand the implications of each decision, fostering a mindset that is prepared for quick, informed responses in actual clinical settings [6, 69–71].

One of the critical benefits of VR in decision-making training is the opportunity it provides for students to practice high-stakes decisions in controlled yet realistic scenarios repeatedly. Healthcare students who participated in VR simulations designed to mimic emergency room situations showed measurable improvements in critical thinking and problem-solving abilities compared to their peers who trained with traditional methods [72–74]. These students were able to apply their learned skills more effectively when faced with similar challenges in clinical practice, suggesting that VR not only improves individual decision-making but also promotes greater situational awareness [75–77].

Furthermore, AI integration enables these simulations to adapt based on students’ actions, promoting an individualized learning experience that challenges students at their level of competence. For example, suppose a student consistently demonstrates proficiency in one aspect of clinical decision-making. In that case, the AI can adjust the simulation to introduce new complexities, such as sudden changes in a patient’s condition, requiring students to reassess and adapt their treatment strategies accordingly. This adaptive feedback approach has been shown to enhance cognitive flexibility—a skill crucial for clinical decision-making under pressure [78–80]. Students trained in AI-augmented VR simulations were better prepared to navigate unexpected clinical changes, essential for maintaining high-quality patient care [78–80].

VR simulations also contribute to ethical decision-making by placing students in scenarios that require them to consider patient values, prioritize care, and balance treatment options. When students were exposed to ethically complex situations within VR—such as decisions regarding life support or resource allocation—they gained practical insight into the ethical dimensions of patient care [81–83]. VR’s immersive nature helps bridge the gap between theoretical ethics education and practical decision-making, allowing students to weigh options and consider the broader implications of their patient-centered actions [6, 84].

Additionally, VR and AI facilitate reflective practice, a crucial component of clinical decision-making. By enabling students to review their choices and receive AI-guided feedback on possible alternatives, VR fosters a learning process that addresses correct actions and explores missed opportunities and areas for improvement. Students who engaged in VR with real-time AI analysis reported improved critical reflection, which is integral to making well-considered clinical decisions [85–87]. Reflective practice within these simulations allows students to assess their strengths and areas needing improvement, building resilience and insight essential for patient-centered care.

VR and AI-enhanced simulations provide a powerful platform for developing clinical decision-making skills in healthcare education. These technologies prepare students to handle diverse clinical situations with greater competence and confidence by simulating real-world challenges and ethical complexities. The tailored feedback from AI in VR scenarios further deepens students’ critical thinking and cognitive adaptability, ensuring they can make informed, patient-centered decisions in practice.

## Discussion

This narrative review set out to explore the combined impact of Artificial Intelligence (AI) and Virtual Reality (VR) on interdisciplinary learning outcomes and patient safety in healthcare education. Our synthesis highlights several key themes: the transformative power of adaptive learning, the depth of immersive skill development, the enhancement of interdisciplinary teamwork, and the significant improvement in patient safety preparedness. This section examines the interpretation of these findings, discusses the overarching contributions to patient safety, acknowledges the limitations of this review, and outlines the critical implications for practice and future research, providing reassurance about the positive impact of AI and VR on patient safety.

### Unpacking the synergistic impact of AI and VR in healthcare education

The integration of AI into VR simulations represents a significant departure from traditional didactic and static simulation methods, fundamentally reshaping the field of healthcare education. In these simulations, AI acts as a dynamic controller, creating adaptive learning pathways that redefine personalized education. This is not merely about individualizing content; it is about optimizing the learning curve and ensuring foundational skills are mastered before progressing to complex challenges [9, 23, 24]. This approach, which continuously assesses learner progress and dynamically adjusts scenario complexity and feedback, directly enhances procedural accuracy and learner confidence [25–27]. Evidence suggests that such tailored experiences are crucial for deeper long-term memory retention and procedural accuracy, directly impacting a learner’s readiness for real-world clinical demands and reducing the chances of error upon transition to practice (Elendu et al., 2024; Halkiopoulos & Gkintoni, 2024; Zhai et al., 2021; Seaba, 2023). The real-time feedback inherent in AI systems during VR simulations plays a significant role in helping students internalize best practices, fostering a proactive mindset essential for swift and accurate responses in critical situations [29, 30]. Ultimately, AI-enabled adaptive learning in VR simulations aligns educational content with individual learning needs, thereby empowering students with the confidence and ability to manage complex patient care scenarios, leading to a more precise, resilient, and patient-focused generation of healthcare professionals [6, 35–39].

Complementing this, immersive VR environments provide an unparalleled platform for the hands-on development of critical clinical skills. This review highlights the unique capacity of VR to simulate complex medical scenarios with high fidelity, creating a realistic and risk-free environment where students can engage deeply with intricate tasks, such as surgical procedures or emergency responses. For instance, in a surgical procedure simulation, students can practice making incisions and suturing in a highly realistic virtual environment (Lavoie et al., 2024; Pottle, 2019). This experiential approach fosters a profound level of skill acquisition and competency, surpassing the limits of traditional training methods. A key analytical insight is VR’s ability to replicate high-stakes scenarios—such as cardiac arrest situations—that healthcare students rarely encounter in conventional training yet are paramount for patient safety. The reviewed literature demonstrates that repeated, high-fidelity practice in these immersive environments significantly boosts students’ confidence and accuracy in performing critical procedures CPR [41–43]. This enhanced confidence and proficiency, cultivated in a safe setting, directly translates to improved clinical performance and better skill retention in real-world scenarios.

VR simulations are not just about technical skills; they play a pivotal role in enhancing decision-making and situational awareness. By immersing students in high-stakes choices within a controlled yet realistic environment, VR fosters quick and accurate judgment in diagnosing conditions and selecting treatment pathways. This form of decision-making under pressure is a crucial aspect of clinical training, as rapid, sound decisions directly impact patient outcomes. The immersive qualities also contribute to increased engagement and reduced anxiety during training, allowing learners to focus more clearly and make more thoughtful decisions—a vital skill for clear thinking under pressure in healthcare. This emphasis on the role of VR in decision-making and situational awareness instills confidence in the audience about the effectiveness of these simulations.

### Interdisciplinary teamwork: a core element of patient safety

The combination of Virtual Reality (VR) and Artificial Intelligence (AI) in healthcare education represents a significant advancement in training professionals to work cohesively in interdisciplinary teams. Effective collaboration across diverse healthcare disciplines, including nursing, medicine, and allied health professions, is essential for delivering comprehensive patient care. VR provides a realistic and engaging platform for students to practice team-based scenarios, replicating the dynamic and multifaceted nature of clinical settings. Critically, AI further enhances these simulations by adapting the scenarios in response to team interactions, prompting students to overcome real-time challenges together [46].

This review underscores the substantial benefits of VR-based teamwork simulations in improving communication and decision-making among healthcare students. Learners engaging in VR simulations focused on acute trauma cases, which require fast-paced decision-making and clear, coordinated communication, report a clearer understanding of each team member’s role. This insight enables better coordination and faster response times under pressure, directly preparing them for high-stakes, interdisciplinary teamwork.

Moreover, these interdisciplinary VR simulations actively promote awareness of role specialization—a critical component of effective healthcare teamwork. By allowing students to experience the unique contributions of each role, VR enhances their understanding of how different disciplines intersect to deliver patient-centered care. Students participating in simulations that involve a mix of professional roles tend to express a greater appreciation for interprofessional collaboration, which fosters mutual respect and reliance among team members [4, 50, 51]. This ability to acknowledge and incorporate diverse expertise is vital for ensuring comprehensive patient care in actual healthcare settings.

A particularly compelling aspect is how AI-enhanced VR introduces unplanned, evolving challenges within simulations—such as sudden changes in a patient’s condition or unexpected complications. This feature requires teams to reassess and adapt their strategies quickly, fostering adaptive expertise. The reviewed literature suggests that VR training enables students to develop the flexibility and quick thinking necessary to navigate complex, unpredictable situations, thereby improving their readiness to handle real-life uncertainties. This adaptability is essential for patient safety, as effective teamwork and clear communication can prevent adverse events by enabling healthcare teams to respond cohesively to sudden challenges [47, 51–53].

Ultimately, VR and AI create a robust environment for interdisciplinary teamwork training in healthcare education. By facilitating team-based clinical simulations, these technologies enable students to build essential collaborative skills, significantly enhancing their preparedness for the complexities of modern healthcare environments. This innovative approach to training not only bolsters individual competencies but also cultivates a coordinated, team-oriented mindset, which is critical to achieving high-quality patient outcomes and ensuring patient safety.

### Enhancing clinical judgment, ethical Decision-Making, and overall patient safety preparedness

Building upon the individual strengths of adaptive learning, immersive skill development, and enhanced interdisciplinary teamwork, the combined application of AI and VR significantly elevates patient safety preparedness across the spectrum of healthcare education, particularly by sharpening clinical judgment and ethical reasoning.

Integrating AI with VR effectively enhances students’ clinical decision-making skills, providing a robust foundation for making critical and timely decisions in complex patient care scenarios. Through VR simulations, students experience realistic patient interactions and medical conditions, enabling them to engage in diagnostic reasoning and treatment planning within a safe and controlled environment. This setup enables learners to understand the implications of each decision with AI-driven feedback that guides them toward informed responses in natural clinical settings, fostering confidence and accuracy—a fundamental aspect of patient safety [6, 69–71].

A significant advantage of VR for decision-making training is the repetitive practice it allows, particularly in high-stakes scenarios. Students participating in VR simulations that mimic emergency room situations demonstrate measurable improvements in critical thinking and problem-solving abilities, often outperforming their peers who were trained through traditional methods in applying these skills during real clinical challenges. This enhances individual decision-making and fosters greater situational awareness in urgent care settings [75–77]. The adaptability of AI-integrated VR simulations further promotes this by introducing challenges at an appropriate level of complexity, requiring students to re-evaluate and adapt their treatment strategies in response to dynamic patient conditions, thereby enhancing cognitive flexibility crucial for high-pressure clinical environments [78–80].

In addition to practical skills, VR simulations provide a powerful avenue for developing ethical decision-making abilities by immersing students in scenarios that necessitate consideration of patient values, care prioritization, and complex ethical dilemmas. Students exposed to VR-based ethical challenges, such as decisions surrounding life support or resource allocation, gain profound insights into the ethical dimensions of patient care (Elendu et al., 2024; Amugongo et al., 2023; Andersson et al., 2022; Steele et al., 2020; Gagne & C, 2023). The immersive quality of VR helps bridge the gap between theoretical ethical principles and practical application, allowing students to weigh options and make patient-centered decisions grounded in ethical considerations.

Furthermore, AI and VR also support reflective practice, a critical component of refined decision-making. By providing real-time feedback on their choices and allowing exploration of alternative responses, VR encourages students to reflect on their actions, helping them build the resilience and insight essential for clinical environments. Students engaging in VR simulations with AI-guided feedback report improved reflective practice, which they identify as integral to enhancing their clinical judgment and self-assessment skills, fostering continuous learning [85–87]. This continuous improvement in judgment and self-assessment directly contributes to overall patient safety.

The ability to practice complex procedures without risking patient harm, coupled with real-time, AI-driven feedback for immediate error correction, directly reduces learner error rates. This cultivates not only technical competence but also instills a proactive approach to risk mitigation. VR simulation training has been shown to boost students’ confidence in handling clinical procedures and patient interactions—a vital factor for patient safety. Healthcare professionals who are self-assured in their skills tend to exhibit greater accuracy and resilience under pressure, reducing the likelihood of errors [62–64]. Moreover, VR simulations with real-time error recognition and AI-enhanced feedback encourage students to adopt a reflexive approach to patient care. By incorporating various “what-if” scenarios, VR allows students to explore potential outcomes based on their decisions, enhancing their awareness of risk management. Students who participated in VR-based training were less likely to encounter critical safety incidents when entering clinical practice, attributing this to a heightened ability to anticipate and address safety risks. This proactive training approach enables future healthcare providers to internalize a mindset focused on patient safety and risk prevention, fostering a culture of care that prioritizes patient well-being [65–68].

Ultimately, AI- and VR-enhanced simulations are profoundly shaping healthcare education by cultivating students’ clinical decision-making abilities, ethical reasoning, and overall preparedness for patient safety. These technologies prepare students to handle various clinical situations confidently and skillfully by providing realistic scenarios and ethical complexities. The tailored feedback AI offers within VR simulations further sharpens critical thinking and adaptability, ensuring students are well-prepared to deliver informed, patient-centered care, supporting safe patient outcomes, and building a competent, resilient workforce poised to face the evolving challenges of modern healthcare.

### The role of emerging technologies in fostering interdisciplinary collaboration and enhancing patient safety

The integration of Artificial Intelligence (AI) and Virtual Reality (VR) in healthcare education offers transformative potential to foster interdisciplinary collaboration and enhance patient safety outcomes. AI-powered virtual agents within VR simulations have proven effective in replicating the dynamics of interprofessional teams. For instance, Liaw et al. (2023) demonstrated that an AI-powered doctor in VR sepsis training matched the performance of human-controlled simulations and significantly improved nursing students’ clinical knowledge—highlighting AI’s scalability benefits where human resources are constrained [46]. This scalability suggests a future where AI can bridge the gap in healthcare education, making it more accessible and effective. Furthermore, immersive VR simulations consistently improve team communication and collaboration: nursing students using IVRS exhibited marked gains in role clarity and assertive communication when practicing protocols like ISBAR (Introduction, Situation, Background, Assessment, Recommendation) and CUS (*I am Concerned*,* I am Uncomfortable*,* This is a Safety issue)*, fostering safer clinical environments [88]. Broader reviews support this trend, indicating that AI-enhanced and VR-enabled simulation platforms significantly enhance interprofessional collaboration, decision-making, and patient safety competencies in healthcare education. Additionally, prospective applications of AI-driven VR—for example, in surgical team training—suggest that real-time performance analytics and adaptive feedback could standardize competency assessments and maintain high-quality care across clinical disciplines [89]. Collectively, this literature suggests that the synergy of AI and VR creates an immersive, data-driven learning environment that enhances teamwork, clinical reasoning, and procedural safety—core pillars for equipping healthcare professionals to meet the complex demands of modern patient care.

### The distinct contribution of dynamically adaptive team simulations

Beyond enhancing individual skills and fostering general collaboration, a particularly novel and impactful insight from this review is the emergence of AI-enhanced VR simulations that dynamically adapt to team interactions [46, 90]. Unlike static team scenarios, these advanced platforms leverage AI to analyze real-time communication, leadership, and decision-making patterns within a virtual team, then *adjust the simulation’s complexity or challenges in response to the team’s collective performance*. This capability extends beyond simply practicing together; it actively shapes team dynamics, prompting learners to refine their adaptive problem-solving and interprofessional responsiveness in an organic, high-fidelity environment. This unique feature, as highlighted by the reviewed literature, represents a significant leap forward, as it directly addresses the fluid and unpredictable nature of real-world clinical teamwork. Emphasizing this dynamic adaptability underscores a critical, often overlooked, dimension of readiness that is paramount for complex patient safety scenarios and offers a particularly promising avenue for future research and advanced healthcare training.

### Limitations of the narrative review

While this narrative review synthesizes current insights into the combined impact of AI and VR on interdisciplinary learning and patient safety in healthcare education, it is essential to acknowledge certain limitations inherent to its methodology. As a narrative review, it does not employ the systematic search and rigorous appraisal protocols characteristic of systematic reviews or meta-analyses. Consequently, the synthesis of evidence presented here reflects a qualitative overview rather than a comprehensive, statistically pooled analysis, which may limit the generalizability and quantitative strength of the conclusions. Furthermore, the selection of studies might be subject to publication bias, where studies reporting positive or significant findings are more likely to be published and subsequently included, potentially skewing the perception of efficacy. The absence of meta-analytic data, in particular, means that specific effect sizes or aggregated statistical conclusions regarding the impact of these technologies cannot be provided. Therefore, while this review offers valuable conceptual insights and identifies key themes, readers should interpret the findings with an understanding of these methodological constraints.

### Implications for practice and future research

The findings of this review carry significant implications for the future of healthcare education and the broader goal of patient safety. For **educational institutions and curriculum developers**, there is a clear imperative to move beyond traditional methods and integrate AI-enhanced VR simulations as core components of training programs. This includes investing in the necessary technological infrastructure, ensuring robust system maintenance and scalability, and developing tailored, adaptive learning modules for diverse disciplines.

For faculty, this shift necessitates dedicated professional development programs focused on leveraging AI and VR effectively. Educators must be equipped not only to operate these technologies but also to design compelling learning experiences. This emphasis on the role of educators in the future of healthcare education guides the reader’s expectations and highlights the importance of their role in this shift.

Regarding **future research**, several avenues emerge. While this review highlights promising applications, robust longitudinal studies are essential to conclusively determine the long-term impact of AI and VR training on actual patient outcomes, particularly in terms of measurable error reduction in clinical settings. Research should also focus on establishing clear benchmarks for these outcomes, conducting cost-effectiveness analyses of large-scale implementations, and exploring the ethical implications of increasingly sophisticated AI integration. Furthermore, studies investigating the optimal balance between adaptive individual learning and dynamic team-based simulations, as well as the best way to integrate these into existing curricula, would be highly beneficial. Continued exploration of specific error types mitigated by these technologies would also enrich the evidence base.

In conclusion, the combined power of AI and VR is not just a tool but a transformative force poised to reshape healthcare education fundamentally. It offers unprecedented opportunities for personalized, immersive, and collaborative learning. By understanding and strategically addressing the implications outlined here, the field can collectively advance toward a future of safer and more effective patient care. This emphasis on patient safety underscores the urgency and importance of the research and development in this field.

## Recommendations

Based on the findings and comprehensive analysis from this narrative review on the combined impact of Artificial Intelligence (AI) and Virtual Reality (VR) on interdisciplinary learning outcomes and patient safety in healthcare education, several vital recommendations emerge. These aim to maximize the potential benefits of AI and VR in fostering critical skills in teamwork, decision-making, and patient safety, directly leveraging the insights gained from the reviewed literature.

### For healthcare institutions and educational bodies

These recommendations focus on the strategic implementation and integration of AI and VR within educational curricula and infrastructure, informed by the identified benefits of immersive and adaptive learning.

#### Develop comprehensive, adaptive training programs

Healthcare institutions and educational bodies should prioritize the implementation of adaptive, AI-driven virtual reality (VR) simulations. As this review highlights their effectiveness, these programs should dynamically evolve in complexity based on individual learner progression and be tailored to specific disciplines while promoting interdisciplinary collaboration through shared, realistic case scenarios. By tailoring challenges to individual competence levels, AI can ensure that learners remain engaged and gain practical expertise across a wide range of clinical situations, reflecting the personalized learning approach observed in the literature.

#### Standardize VR simulations for ethical and situational awareness

It is crucial to integrate standardized VR scenarios that simulate ethically complex situations, as explored in this review’s analysis of decision-making. Such integration would prepare students for real-life moral dilemmas they may encounter in clinical practice. Incorporating standardized ethical modules across healthcare curricula would enhance consistency in ethical training and better equip students to navigate complex moral choices in patient care, a key aspect identified as essential for patient safety preparedness.

#### Incorporate interdisciplinary Team-Based training to enhance collaboration

Given the crucial role of interdisciplinary teamwork in modern healthcare, as affirmed by the reviewed studies, VR and AI simulations should prioritize team-based exercises that allow nursing, medicine, and allied health students to practice collaborative decision-making. The review’s findings suggest that immersing students in realistic, high-pressure environments where effective teamwork is required can significantly improve communication and decision-making skills. These simulations help participants understand and respect the roles of other disciplines, promoting an integrative approach to patient care that directly mirrors real-world demands.

#### Foster faculty development programs in AI and VR technologies

To successfully implement and sustain AI and VR in healthcare curricula, as indicated by the need for effective integration, it is essential to provide faculty with the training and resources needed to design, evaluate, and manage these technologies effectively. Instructor preparedness is critical to maximizing the educational impact of new technologies. Therefore, developing professional development programs focused on VR and AI applications in healthcare education would equip educators with the skills to guide students through these complex simulations, ensuring compelling learning experiences that capitalize on the technologies’ potential.

### For researchers

These recommendations highlight areas for future investigation, building directly on the identified gaps and insights from this narrative review to foster a more robust evidence base.

#### Expand research to measure longitudinal impacts on patient outcomes

Further research should rigorously explore the long-term impact of AI and VR training on actual patient outcomes. While this review’s findings suggest promising avenues for enhanced patient safety, robust longitudinal studies are crucial to conclusively determine the extent to which these interventions lead to measurable error reduction in clinical settings. These studies are necessary to evaluate the retention and application of skills over time in complex clinical situations, thereby strengthening the evidence base for sustained effectiveness.

### For collaborating stakeholders and policy makers

These recommendations focus on broader systemic changes and partnerships stemming from the implications drawn regarding widespread adoption and efficacy.

#### Establish regular feedback loops using AI analysis

AI’s capacity for real-time feedback provides unique opportunities for continual learning and improvement in clinical skills, as demonstrated by enhanced performance in simulations. Therefore, integrating AI-based assessment tools within VR simulations should be prioritized to allow students to receive immediate, actionable feedback on their performance. Immediate feedback enhances learning outcomes by enabling students to correct mistakes promptly. It solidifies their understanding of the impact of their actions in clinical settings, thereby maximizing the learning potential identified in this review.

#### Encourage institutional collaboration to share best practices and resources

By collaborating with other institutions and sharing best practices in AI and VR, as this review highlights, the collective benefit can lead to a more efficient and cost-effective implementation of these technologies across the field. Partnerships allow institutions to pool resources and experiences, providing a standardized approach to AI and VR integration that benefits healthcare education broadly. A collaborative approach can facilitate the development of high-quality, shared virtual reality (VR) simulations tailored to various medical and nursing disciplines, promoting a uniform standard in clinical education.

Implementing these recommendations, directly informed by the synthesized evidence in this review, would significantly enhance the efficacy and reach of AI and VR in healthcare education. This will prepare students with the critical interdisciplinary skills and patient-centered competencies necessary for modern clinical practice. By continuously refining VR and AI training modules based on evidence-based practices and fostering robust institutional collaboration, healthcare education can better equip future professionals to deliver safe, high-quality care.

## Conclusion

The study “Exploring the Combined Impact of Artificial Intelligence and Virtual Reality on Interdisciplinary Learning Outcomes and Patient Safety in Healthcare Education” highlights the significant potential of these technologies to elevate educational practices within the healthcare field. By integrating Artificial Intelligence (AI) and Virtual Reality (VR), healthcare education has experienced transformative improvements in critical areas, including interdisciplinary teamwork, clinical decision-making, patient safety preparedness, and ethical competency. VR’s immersive environments enable students to engage in realistic simulations that replicate high-stakes clinical scenarios, promoting experiential learning and critical skill development without exposing them to patient risk. Combined with AI, these simulations provide dynamic, real-time feedback and adaptive challenges that support individualized learning pathways, fostering cognitive flexibility and practical expertise.

This study highlights that when AI and VR are used synergistically, they enhance students’ technical skills and refine their communication, ethical reasoning, and decision-making abilities. Such comprehensive training in a controlled yet realistic setting prepares future healthcare professionals to respond effectively to real-world clinical challenges, improving patient safety and care quality. The combined use of AI and VR represents a forward-thinking approach to healthcare education, equipping students with essential competencies and confidence to navigate the complexities of modern patient-centered care.

## Electronic supplementary material

Below is the link to the electronic supplementary material.

Supplementary Material 1

Supplementary Material 2

Supplementary Material 3

Supplementary Material 4

Supplementary Material 5

Supplementary Material 6

Supplementary Material 7

Supplementary Material 8

## Data Availability

No datasets were generated or analysed during the current study.

## Declarations

### Compliance with the helsinki declaration

This study was conducted in accordance with the principles of the Declaration of Helsinki.
