# Supplementary material for: The combined impact of AI and VR on interdisciplinary learning and patient safety in healthcare education: a narrative review
Source: BMC Med Educ. 2025 Jul 11;25:1039. doi: 10.1186/s12909-025-07589-7 (PMC12254989; doi:10.1186/s12909-025-07589-7)
Supplement: Supplementary file 1 — Supplementary Material 1 [file 12909_2025_7589_MOESM1_ESM.docx]

**Figure 1:** PRISMA flow diagram

N=76 Studies included in the analysis: (*n1=17), (n2=10), (n3=10), (n4=18), and (n5=21)*

Studies included in review synthesis (*n=118*)

Full-text articles excluded, with reasons such as design, languages,populations, missing information and s(*n=450*)

Number of full-text articles assessed for eligibility (*n=568*)

Number of obviously irrelevant and duplicate reports excluded for not fulfilled the selection criteria and other constraints *(n= 1082)*

Number of titles (and/or abstracts) screened (*n=1650)*

Number of records *(n=6028)*

Number of additional records identiﬁed through searching other sources (*n =89*)

Number of records identiﬁed through searching electronic databases (*n =*5939)

Identification

Included

Screening

Eligibility

**Source:** The authors
