# Supplementary material for: The combined impact of AI and VR on interdisciplinary learning and patient safety in healthcare education: a narrative review
Source: BMC Med Educ. 2025 Jul 11;25:1039. doi: 10.1186/s12909-025-07589-7 (PMC12254989; doi:10.1186/s12909-025-07589-7)
Supplement: Supplementary file 2 — Supplementary Material 2 [file 12909_2025_7589_MOESM2_ESM.docx]

**Table 1: Illustration on how articles were analyzed**

| **Category** | **Subcategory** | **Themes** | **Authors and Year of Publication** |
| --- | --- | --- | --- |
| **Main focus area in the research or educational intervention** (e.g., "Patient Safety Preparedness") | **Specific aspect within the main category** (e.g., "Error Recognition and Correction") | **Summary of findings or insights related to the category and subcategory** (e.g., "AI-driven feedback helps identify and correct errors in real-time, enhancing patient safety skills") | **List of authors with publication year** (e.g., " Hong et al., 2023") |
