# Supplementary material for: The combined impact of AI and VR on interdisciplinary learning and patient safety in healthcare education: a narrative review
Source: BMC Med Educ. 2025 Jul 11;25:1039. doi: 10.1186/s12909-025-07589-7 (PMC12254989; doi:10.1186/s12909-025-07589-7)
Supplement: Supplementary file 3 — Supplementary Material 3 [file 12909_2025_7589_MOESM3_ESM.docx]

Table 6: **Ethical and Psychological Impact**

| **Category** | **Subcategory** | **Findings** | **Authors** |
| --- | --- | --- | --- |
| **Ethical and Psychological Impact** | **Psychological Readiness for High-Stakes Environments** | Exposure to realistic simulations builds resilience and stress management skills in high-pressure situations. | Božić, 2024;  Elendu et al., 2024; George, 2023; Strielkowski et al., 2024 |
| **Ethical and Psychological Impact** | **Ethical Awareness in Simulations** | VR simulations present ethical dilemmas, fostering critical thinking around patient safety and care ethics. | Datta et al., 2012;  Okuda et al., 2009;  Wood et al., 2022; Abdellatif et al., 2022; Scherr et al., 2023;  Xu et al., 2024;  Apostolakis et al., 2022; Langer & Landers, 2021; Munir et al., 2022 |
| **Ethical and Psychological Impact** | **Mental Health and Simulation Fatigue** | Understanding the psychological effects of repeated high-stress scenarios in VR on students’ well-being. | Amugongo et al., 2023; Andersson et al., 2022; Steele et al., 2020. Elendu et al., 2024; Gagne & C, 2023  Dai & Ke, 2022; Hooda et al., 2022; Ouyang et al., 2023 |
