# Supplementary material for: The combined impact of AI and VR on interdisciplinary learning and patient safety in healthcare education: a narrative review
Source: BMC Med Educ. 2025 Jul 11;25:1039. doi: 10.1186/s12909-025-07589-7 (PMC12254989; doi:10.1186/s12909-025-07589-7)
Supplement: Supplementary file 4 — Supplementary Material 4 [file 12909_2025_7589_MOESM4_ESM.docx]

Table 4: **Interdisciplinary Teamwork**

| **Category** | **Subcategory** | **Findings** | **Authors** |
| --- | --- | --- | --- |
| **Interdisciplinary Teamwork** | **Team-Based Simulation** | AI and VR enable interdisciplinary team simulations, enhancing collaborative skills and patient safety. | S. Y. Liaw et al., 2023  (S. Y. Liaw et al., 2023)  A. Hamilton, 2024  Steen et al., 2024 |
| **Interdisciplinary Teamwork** | **Enhanced Communication and Decision-Making** | VR scenarios improve team communication and decision-making under pressure. | Shorey et al., 2020  Grassini & Ratcliffe, 2024  Kim et al., 2024;  S. Liaw et al., 2020;  Qiao et al., 2021) |
| **Interdisciplinary Teamwork** | **Role Specialization Training** | Students understand and respect roles within a team, crucial for effective patient-centered care. | Hamilton et al., 2021;  Lateef, 2010; S. Liaw et al., 2020  Elendu et al., 2024;  Pottle, 2019; Wang & Li, 2024 |
