# Supplementary material for: The combined impact of AI and VR on interdisciplinary learning and patient safety in healthcare education: a narrative review
Source: BMC Med Educ. 2025 Jul 11;25:1039. doi: 10.1186/s12909-025-07589-7 (PMC12254989; doi:10.1186/s12909-025-07589-7)
Supplement: Supplementary file 5 — Supplementary Material 5 [file 12909_2025_7589_MOESM5_ESM.docx]

**Table 2: Adaptive Learning**

| **Category** | **Subcategory** | **Findings** | **Authors** |
| --- | --- | --- | --- |
| **Adaptive Learning** | **AI-driven Personalization** | **AI tailors VR simulations to individual performance, enhancing skill acquisition and addressing weaknesses.** | Gallagher et al., 2005  Giannakos et al. 2024;  Liu et al., 2023  Chan & Zary, 2019; Esmaeilzadeh, 2024;  Halbig et al., 2022 |
| **Adaptive Learning** | **Customized Feedback** | **AI provides real-time, targeted feedback, improving students’ skills and awareness of patient safety risks.** | Feng, 2024;  Lin et al., 2024;  ZABOT, 2024;  Seaba, 2023 |
| **Adaptive Learning** | **Skill Retention** | **Adaptive learning improves long-term retention and transferability of skills to clinical settings.** | Elendu et al., 2024;  Halkiopoulos & Gkintoni, 2024;  Zhai et al., 2021;  Ahmed et al., 2010;  Carlson, 2023;  Cascella et al., 2023;  Mirchi et al., 2020; Xie et al., 2021 |
