# Supplementary material for: The combined impact of AI and VR on interdisciplinary learning and patient safety in healthcare education: a narrative review
Source: BMC Med Educ. 2025 Jul 11;25:1039. doi: 10.1186/s12909-025-07589-7 (PMC12254989; doi:10.1186/s12909-025-07589-7)
Supplement: Supplementary file 6 — Supplementary Material 6 [file 12909_2025_7589_MOESM6_ESM.docx]

Table 5: **Patient Safety Preparedness**

| **Category** | **Subcategory** | **Findings** | **Authors** |
| --- | --- | --- | --- |
| **Patient Safety Preparedness** | **Error Recognition and Correction** | Real-time feedback helps students identify and correct errors, reducing potential patient risks. | Hong et al., 2023;  Lukashova-Sanz et al., 2023;  Rashid & Kausik, 2024;  Rasouli et al., 2024 |
| **Patient Safety Preparedness** | **Simulation of Ethical Scenarios** | VR offers practice in ethically challenging scenarios, preparing students for moral decision-making. | Bachmann et al., 2022;  Motola et al., 2013;  Thakkar et al., 2024 Aggarwal et al., 2010; Ashcroft et al., 2021; Hamdi & Al Thobaity, 2023. |
| **Patient Safety Preparedness** | **Confidence in Patient Care** | Practice in VR builds student confidence in performing procedures accurately, supporting patient safety. | King et al., 2018;  Li et al., 2018;  Orbæk et al., 2015; Paranjape et al., 2019. |
