# Supplementary material for: The combined impact of AI and VR on interdisciplinary learning and patient safety in healthcare education: a narrative review
Source: BMC Med Educ. 2025 Jul 11;25:1039. doi: 10.1186/s12909-025-07589-7 (PMC12254989; doi:10.1186/s12909-025-07589-7)
Supplement: Supplementary file 7 — Supplementary Material 7 [file 12909_2025_7589_MOESM7_ESM.docx]

**Table 7: Common AI Models and Examples**
*Adapted from Klingler (2023), Qiu et al. (2023), and recent advancements*

| **AI Model Type** | **Examples** |
| --- | --- |
| **Transformer Models** | GPT-3, GPT-4, GPT-4o, BERT, RoBERTa, T5, XLNet, Claude, Gemini |
| **Generative Adversarial Networks (GANs)** | StyleGAN, CycleGAN, BigGAN |
| **Convolutional Neural Networks (CNNs)** | ResNet, VGG, Inception, EfficientNet, DenseNet |
| **Recurrent Neural Networks (RNNs)** | LSTM, GRU, Bi-LSTM |
| **Reinforcement Learning (RL) Models** | Q-learning, Deep Q-Network (DQN), AlphaGo, MuZero |
| **Decision Trees and Ensemble Models** | Random Forest, XGBoost, LightGBM, Gradient Boosting Machines (GBM) |
| **Clustering & Dimensionality Reduction** | K-means, Principal Component Analysis (PCA), t-SNE, UMAP |
| **Multimodal Models** | CLIP, DALL·E, Flamingo, GPT-4o, Gemini |
