# Supplementary material for: The combined impact of AI and VR on interdisciplinary learning and patient safety in healthcare education: a narrative review
Source: BMC Med Educ. 2025 Jul 11;25:1039. doi: 10.1186/s12909-025-07589-7 (PMC12254989; doi:10.1186/s12909-025-07589-7)
Supplement: Supplementary file 8 — Supplementary Material 8 [file 12909_2025_7589_MOESM8_ESM.docx]

Table 3: **Immersive Skill Development**

| **Category** | **Subcategory** | **Findings** | **Authors** |
| --- | --- | --- | --- |
| Immersive Skill Development | High-Fidelity Clinical Simulations | VR offers realistic practice of complex procedures, improving practical skill and confidence. | Lavoie et al., 2024; Pottle, 2019. |
| Immersive Skill Development | Simulation of High-Stakes Scenarios | Students experience critical patient-care scenarios safely, preparing them for real-life emergencies. | Jacobs & Maidwell-Smith, 2022; Rushton et al., 2020; Trevi et al., 2024; |
| Immersive Skill Development | Repetitive Skill Building | Students can practice repeatedly in VR, reinforcing essential clinical skills without patient risk. | Bani salameh et al., 2023; Liu et al., 2023. |
